# Premature Destruction of Microbubbles during Voiding Urosonography in Children and Possible Underlying Mechanisms: Post Hoc Analysis from the Prospective Study

**DOI:** 10.1155/2016/1764692

**Published:** 2016-11-20

**Authors:** Maciej Piskunowicz, Dominik Swieton, Dorota Rybczynska, Arkadiusz Szarmach, Edyta Szurowska, Menno Pruijm

**Affiliations:** ^1^Department of Radiology, Medical University of Gdansk, Gdańsk, Poland; ^2^Department of Nephrology and Hypertension, CHUV, Lausanne, Switzerland

## Abstract

The aim of this study is to describe premature microbubbles destruction with contrast-enhanced voiding urosonography (ce-VUS) in children using 2nd-generation ultrasound contrast agents (UCA) and to hypothesize about the reason. 141 children (61 females and 80 males) were included in the study, with mean age of 3.3 years (range 4 weeks–16.0 years), who underwent ce-VUS examination between 2011 and 2014. Premature destruction of the microbubbles in the urinary bladder during ce-VUS was observed in 11 children (7.8%). In all these cases the voiding phase of ce-VUS examination could not be performed because of destroyed UCA microbubbles. This was noted in anxious, crying infants and children with restricted voiding. The premature destruction of ultrasound contrast agent during ce-VUS is an underreported, important limitation of ce-VUS, which prevents evaluation of the voiding phase and the establishment of vesicoureteric reflux (VUR). This was particularly noted in crying infants and children.

## 1. Introduction

Contrast-enhanced voiding urosonography (ce-VUS) is the most common application of second-generation ultrasound contrast agents (UCA) in children [1]. The ce-VUS is generally considered as a promising tool with high accuracy to diagnose vesicoureteric reflux (VUR), comparable to standard voiding cystourethrography (VCUG) [2–4]. Some technical limitations concerning visualization of the urethra in ce-VUS have now been mastered [5, 6]. Administration of the microbubbles is in general well tolerated, and the so far reported side effects were mainly linked to the catheterization procedure [1, 7, 8]. To the best of our knowledge, there are no published reports about difficulties that may arise in the case of premature intravesical destruction of microbubbles. In this post hoc analysis, we describe a series of patients in whom this problem occurred and analyzed the mechanisms possibly responsible for this phenomenon.

## 2. Material and Methods

### 2.1. Subjects

Between January 1st, 2011, and December, 31st, 2014, 141 children (61 girls and 80 boys) with the mean age of 3.3 years (range 4 weeks–16.0 years) underwent ce-VUS and were consequently enrolled to the prospective study according to the protocol. From the study cohort, we performed a post hoc analysis based on the selected children with reports of premature microbubbles destruction and control group of children with fully diagnostic ce-VUS. The control group was sex- and age-matched to the group of children with premature microbubbles destruction (28 children; 11 girls, 17 boys; mean age 2.0 years, range 4 weeks–11.0 years). As far as experiences concerning the use of the second-generation UCA in contrast-enhanced voiding urosonography are still limited, all information about the duration of bladder filling, first symptoms of destruction and subtotal/total destruction or any side effects and possible technical problems like crying, problems with voiding, and so forth were collected in study reports of all cases.

### 2.2. Examination

All examinations were performed without sedation. The indication for the examination was in all situations made by the treating pediatrician. Indications were as follows: urinary tract infection, diagnosed in ultrasound dilatation of the urinary excretory system after nephrologist consultation, follow-up of VUR, and neurogenic bladder.

A baseline gray-scale US and ce-VUS examination of the urinary tract were both performed in the supine and prone positions. All US examinations were performed using an Aloka *α*6 device (Aloka, Hitachi-Aloka Medical, Ltd., Japan), using 5.0–8.0 MHz convex and 8.0–12.0 MHz linear probes; however, ce-VUS was done only with the convex probe. During ce-VUS examination, the mechanical index (MI) was between 0.06 and 0.08. The position of the transducer was continuously altered during the ce-VUS examinations to avoid excessive exposure to insonization and destroying microbubbles in one region. All examinations were recorded on digital video clips. In the case of restriction during the voiding phase, short video clips were recorded every 60–90 seconds to prevent microbubbles excessive destruction.

All children were under prophylactic antibiotic therapy in accordance with the nephrologist's recommendation.

Patients were catheterized transurethral under aseptic conditions with a 4 F–14 F feeding tube (Bicakcilar, Turkey) after its lubrication with lidocaine hydrochloride anesthetic gel (Lignocainum U (2%), Jelfa, Poland). After catheterization, the bladder was completely emptied. A plastic bottle containing a mixture of 0.9% NaCl (Solution of sodium chloride 0.9%, Jelfa, Poland) and sulphur hexafluoride (SonoVue®, Bracco, Milan, Italy) (see below) was connected through the drip system to the catheter and placed about 80 cm above the examination table. The total volume of the bladder was estimated with the Koff formula: volume in milliliters = (age in years + 2) × 30 [9]. The filling of the bladder was continued until the child started to feel the urge to void, the calculated volume was reached, or the dripping speed of the infusion fluid slowed down due to back pressure. With the child in the supine position, both kidneys, the bladder, and the lower ureter were scanned alternately during continued filling and while voiding using the contrast-enhanced ultrasound option. The catheter was removed before voiding. Additionally, during the voiding phase the urethra was examined. In the case of total microbubbles destruction, we had to skip the voiding phase and discontinue the examination.

### 2.3. SonoVue Preparation

Three different lots of sulphur hexafluoride were used.

During one examination session, two up to five children were examined. The period between reconstitution of sulphur hexafluoride and the last examination was never longer than 4 hours. The contrast agent (sulphur hexafluoride, SonoVue®) was dissolved in saline (sodium chloride 0.9%, Jelfa, Poland) shortly before each exam under aseptic conditions. In all cases a total of 0.5–1.0 mL of sulphur hexafluoride was added to a 100–250 mL 0.9% saline solution in a plastic bottle) (prewarmed to 32°) with the use of 2 mL luer-lock type syringe and 18 G needle. Before administration, the mixture was gently shaken until it appeared homogenous. When the expected urinary bladder volume was greater than 250 mL, an additional bottle of the solution was prepared.

### 2.4. Elimination Potential Factors of Microbubbles Destruction

To find an explanation for the premature destruction of microbubbles, all possible causes linked to technical aspects of ce-VUS examination were excluded, including a wide range of operator depending factors which could influence on ce-VUS imaging [10, 11]. To ce-VUS examination, we use only low-frequency probes (convex) with dedicated settings and MI 0.07–0.1. The linear probe was used only at the beginning of the examination to scan the kidneys in b-mode option, but not after contrast administration. The probe was never kept in the same position longer than few seconds. The focus zone was always set in the lowest position. The examinations were performed and analyzed by two experienced physicians. No erroneous composition of the SonoVue solution as a potential source of the problem was identified. During every examination session, on average four children received a solution from the same vial of SonoVue and the premature destruction of microbubbles was never observed in more than one child per session. The order of the patients with this phenomenon in each session was casual. Premature destruction of microbubbles occurred in two children who received the microbubbles first, in three children who received the microbubbles as second, in three children who received as third, in two who received as fourth, and in one child who received as fifth.

The microbubbles due to the high impedance difference backscattered the ultrasound wave at the surface and created a detectable signal. Observed in our study loss of the microbubbles signal in urine bladder could have different reasons. Among others, microbubbles could be destroyed during preparation the solution of saline and microbubbles, too low microbubbles level in solution, and unsuitable insonation power during the examination. However, we paid special attention to the procedure of contrast preparation and technic of examination. In all cases with premature microbubble destruction, in the initial phase of examination signal from contrast filling urinary bladder was always satisfactory and fully diagnostic. In all cases, weakening of signal was observed in the course of the examination.

### 2.5. Definitions

Restricted voiding was defined as a period of at least 10 minutes after the removal of the catheter without effective voiding.

Premature destruction of microbubbles was defined as destruction of microbubbles before the voiding phase, leading to the total vanishing of the microbubbles and precluding evaluation of VUR during the ce-VUS voiding phase.

## 3. Results

In the study group, there were no observed side effects after intravesical application of the UCA. In our study, the number of side effects due to the procedure did not differ significantly from data reported in medical literature and were similar to traditional VCUG.

A total of 11 cases (7.8%) presented premature destruction of the microbubbles in the urinary bladder (Table 1). None of these children had a neurogenic urinary bladder or other urinary bladder abnormalities.

**Figure 1:**
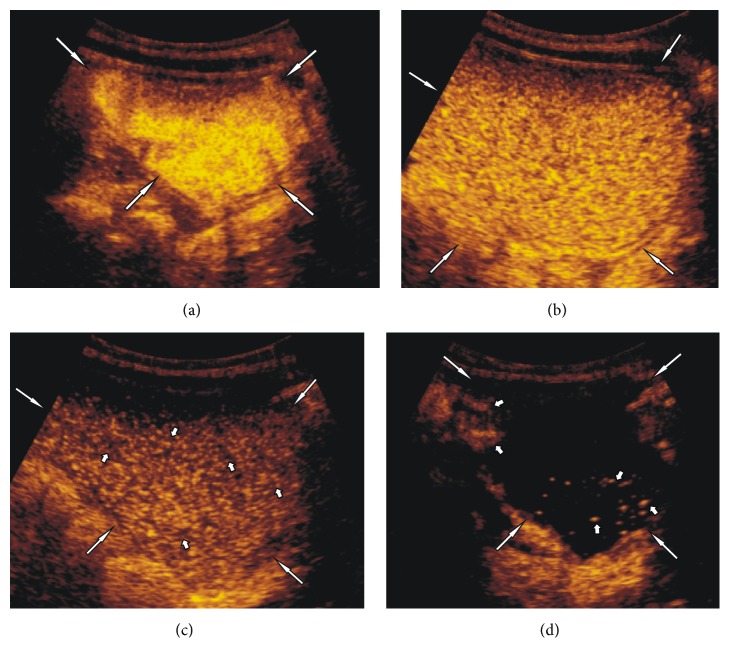
Subject 2: seven-year-old girl. (a) Filling the urinary bladder (white long arrows) with the contrast agent. (b) Urinary bladder (white long arrows) 2 minutes after removal of the catheter and two failed voiding attempts, beginning of microbubbles destruction. (c) Urine bladder (white long arrows) 5 minutes after removal of the catheter with visible areas of destructed microbubbles (short white arrows). (d) Urinary bladder (white long arrows) 6 minutes after removal of the catheter and multiple voiding attempts, with visible single echoes from not destroyed microbubbles (short white arrows).

In the case of three older children (numbers 1, 2, and 5 (Table 1)) the destruction of the microbubbles started 2 or more minutes after complete filling of the urinary bladder and after a few attempts of unproductive voiding (Figures 1(a)–1(d)). All three had the restriction of voiding (likely due to psychogenic stress). The elapsed time to voiding varied from 30 to 45 minutes. Due to earlier destruction of microbubbles (total destruction noticed between 10 and 16 minutes of examination), the assessment of the voiding phase was impossible, and the examination was discontinued.

**Figure 2:**
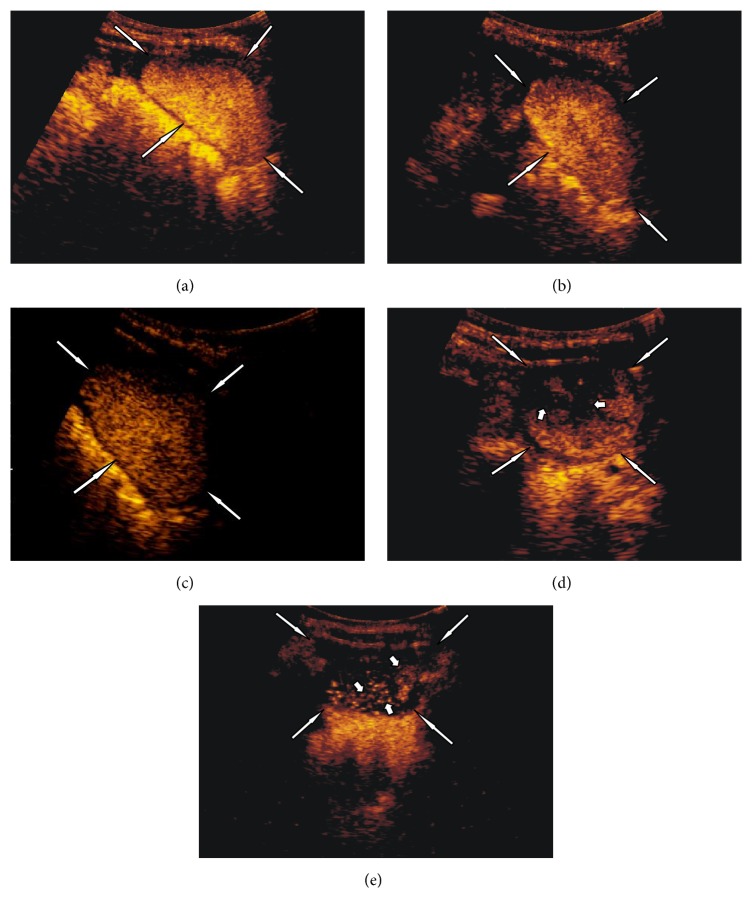
Subject 6: 11-month-old boy, high anxiety and constantly crying during the examination. (a) Filling the urinary bladder (white long arrows) with the contrast agent. (b) Urinary bladder (white long arrows) a few seconds after removal of the catheter, beginning of the microbubbles destruction. (c) Urinary bladder (white long arrows) 1 minute after removal of the catheter. (d) Urinary bladder (white long arrows) 2 minutes after removal of the catheter, with visible areas of absent echoes due to destroyed microbubbles (short white arrows). Urinary bladder (white long arrows) 3 minutes after removal of the catheter with visible single echoes from not destroyed microbubbles (short white arrows).

**Figure 3:**
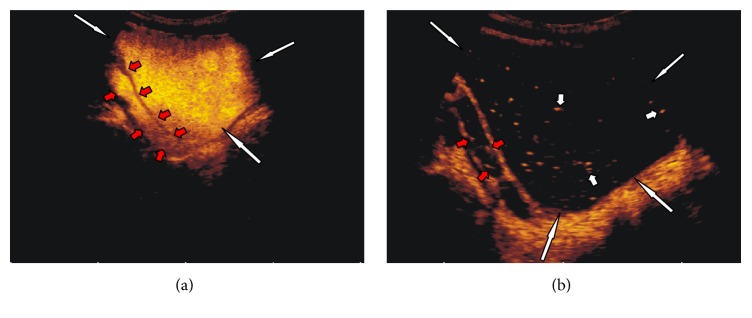
Subject 5: 8-year-1-month-old boy. (a) Urinary bladder (white long arrows) during filling phase. Visible dilated ureter with refluxing microbubbles (red short arrows). (b) Urinary bladder (white long arrows) with single persistent microbubbles of contrast agent 6 minutes after the end of the filling phase (short white arrows). Visible dilated ureter with single refluxing microbubbles (red short arrows).

**Figure 4:**
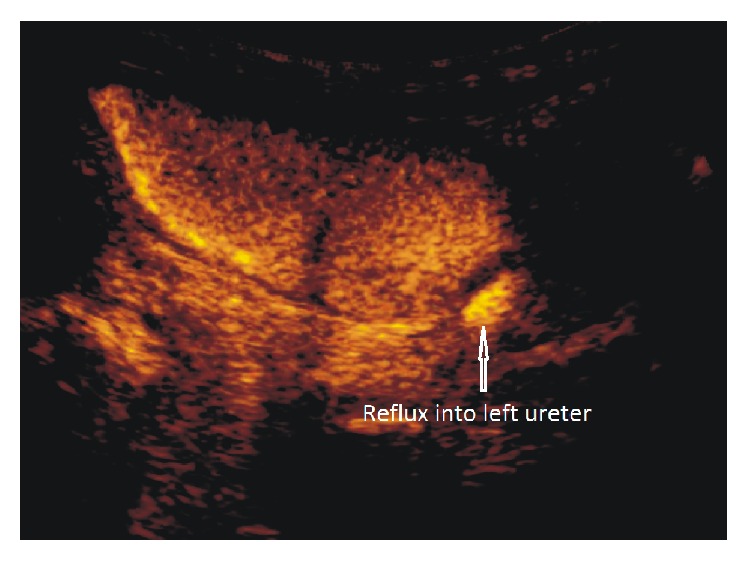
Higher signal of refluxing left ureter than of the urinary bladder, where microbubbles destruction started.

Eight younger children and infants (Table 1) also presented restricted voiding; all of them were anxious and cried without interruption during the entire examination. In this group, the destruction of microbubbles had been noticeable at the end of the urinary bladder filling phase (Figures 2(a)–2(e)). Hence, in three cases (numbers 3, 6, and 9 (Table 1)) an additional dose of saline and contrast solution was administrated before removal of the catheter, but this effort did not resolve the problem and microbubbles continued to be destroyed before the voiding phase. Details concerning the duration of bladder filling and total examination time for cases and controls are given in Table 2. No differences in bladder filling times were seen between the two groups. As expected, total examination time was longer in cases. However, destruction of microbubbles occurred on average five minutes after completion of the bladder filling in the cases, which was within the duration of the total examination time in controls. The evaluation of the voiding phase in all cases of premature contrast destruction was impossible due to the absence of an echo signal from the microbubbles (Figures 3(a) and 3(b)) (Table 1). In cases with vesicoureteric reflux, we observed that in some of them signal in refluxing ureter was strong as at the beginning of the examination, while at the same time weakening of signal due to contrast microbubble destruction in the bladder was evident like in Figure 4.

## 4. Discussion 

Taken together, the results of this monocentric case study suggest that, in 11/141 patients (7.8%), ce-VUS could not be completed due to the premature destruction of the microbubbles. The premature destruction of microbubbles occurred especially in anxious, constantly crying children and in children with restricted voiding and precluded assessing voiding phase during ce-VUS. Consequently, it could have a major impact on the clinical outcome in the case of the ce-VUS procedure.

Therefore, a better understanding of the pathophysiology of this phenomenon is urgently needed.

Destruction of microbubbles occurred on average five minutes after bladder filling. Since this was within the average total examination time of the controls, the destruction of microbubbles cannot simply be ascribed to the longer duration of the examination. None of the controls showed signs of destruction of microbubbles, although their examination time was sometimes up to 2.5 times longer than the destruction time of microbubbles in the case group.

The duration of VUS including the catheterization time and precontrast imaging in our study was approximately 15–20 min. In previous studies, the mean examination time for ce-VUS was between 8 and 34 minutes, depending on the contrast product and ultrasound modality used [4, 6, 12, 13]. Hence, the phenomenon of premature microbubble destruction cannot be explained by a longer examination time in our centre as compared to other groups. Finally, there were no significant differences in age or sex between cases and controls, excluding the possibility of age- or sex-related effect.

The observation that premature destruction occurred in anxious, constantly crying children with prolonged restriction of voiding led us to consider that physical factors might play a role. Hence, it is well known that the “lifetime” and destruction of contrast particles depends on numerous physical factors such as acoustic pressure, hydrostatic pressure, liquid temperature, and the amount of dissolved gas in the ambiance of microbubbles [14–16]. The different factors determining the disappearance time of gas bubbles are given by the equation of Epstein and Plesset, further adapted by de Jong et al. [14, 17]:

(1) dRdtDLCi/Co−1−2σ/RPo−pov/Po1+4σ/3RPo·1R+1πDt,

where *R* is radius of the bubbles, *T* is time, *D* is diffusion constant, *C*_*i*_/*C*_*o*_ is ratio of the dissolved gas concentration to the saturation concentration, *σ* is surface tension, *P*_*o*_ is ambient pressure, *L* is Ostwald coefficient, and *p*_ov_ is overpressure.

This equation shows that the gas bubbles disappear more quickly when they are exposed to overpressure (bolded in the equation). In the case of intravenous administration in clinical practice, an overpressure of microbubbles takes place in two different situations: during their injection (overpressure in the syringe and the needle) and while in the arteries of systemic circulation (systolic blood pressure). As shown by Talu et al., the destruction of perfluorocarbon-filled microbubbles depends on the needle size and injection flow rate [18].

de Jong et al. observed that albumin bubbles tend to shrink and disappear if a hydrostatic pressure of 20 kPa (203.9 cmH_2_O) is applied [14, 19]. Vuille et al. reported that the disappearance time of shelled UCA microbubbles was two, three, and nine times shorter when exposed to pressures of, respectively, 6 kPa (61.2 cmH_2_O), 13 kPa (132.6 cmH_2_O), or 19 kPa (193.7 cmH_2_O) [20].

In vitro experiments suggest that shelled microbubbles have a high resistance to hydrostatic pressure up to 600 mmHg (815.7 cmH_2_O) [16]. However, in vivo studies have illustrated that additional forces such as local shear and forces generated by the transducer acoustic pressure also act on microbubbles and shorten their disappearance time [21].

In the urinary bladder, the intravesical pressure is the sum of pressure induced by detrusor contractions together with the intra-abdominal pressure. The maximum intravesical pressure during voiding recorded by a transurethral catheter in infants and children with no apparent voiding symptoms ranges from 56.6 ± 20.3 cmH_2_O in infants <1 yr to 70.7 ± 13.6 cmH_2_O in 12-year-old children [22]. In another study using suprapubic catheters, intravesical pressure and voiding detrusor pressures varied between 107 and 117 cmH_2_O in infants (3–10 months old); pressures were around 75 cmH_2_O in girls [23]. In infants with lower tract urinary dysfunction characterized by high voiding pressure levels, low bladder capacity, and dyscoordination at voiding, VUR is common with an estimated prevalence around 80% [24]. In infants with known VUR, voiding detrusor pressures ranged between 75 and 234 cmH_2_O in a study by Sillén at al. and 180 cmH_2_O in a study by Chandra and Maddix [24, 25].

The overpressure inside the urinary bladder is potentially a very important factor influencing the disappearance time of gas bubbles. Constant crying and prolonged restriction of voiding are likely to increase, respectively, the abdominal and intravesical pressure. In accordance with the equation of Epstein and Plesset, further adapted by de Jong et al. (see above), the higher the overpressure acting on the microbubbles in the urinary bladder, the faster their destruction. High hydrostatic pressure might be the mechanism responsible for the premature destruction of microbubbles. However, in this post hoc analysis, intravesical and intra-abdominal pressures were not measured; more research is therefore needed to explore further this pathway.

This post hoc analysis has several other limitations. No data were acquired on the composition of the urine on the day of the exam, although the presence of microscopic hematuria or the osmolality might have played a role in the premature destruction of microbubbles.

Besides, data concerning behavior was not available in all examined children. We have prospectively analyzed behavior during voiding urosonography in another group of 35 children undergoing VCUG. We noticed anxiety and crying in 4/16 (25%) of children under 1 years old, in 5/11 (45%) of children between 1 and 4 years, and 1/8 (12%) of children over 4 years old. In none of them, the premature destruction of microbubbles occurred. These percentages were thus largely inferior to the one observed in our cases and strengthen (but not prove) our hypothesis that crying plays a role.

The authors performed another study (in press) comparing classical VCUG and ce-VUS. The agreement between the ce-VUS and VCUG in the diagnosis or exclusion of the VUR reached more than 95% (personal communication). In 7/84 cases, the diagnosis with the ce-VUS was impossible to be set due to damaging microbubbles of contrast; two children were continuously crying while in five cases there were problems with getting voiding phase (personal communication). The frequency of microbubbles destruction was comparable in both studies (personal communication).

## 5. Conclusions

We describe for the first time the presence of premature microbubble destruction in ce-VUS, with an estimated prevalence of 7.8%. As it was post hoc analysis study more epidemiological data are necessary to estimate its prevalence in different populations and countries. According to our assumptions, constantly crying infants and children with restriction during the voiding phase are at risk, probably due to pressure-related mechanisms, and should be under careful observation during the ce-VUS examination. Further research is needed to disentangle the mechanisms responsible for this phenomenon.

All values of the pressure were converted in this text to the unit of cmH_2_O accordingly to the equations as below:

10-millimeter mercury (0°C) = 13.595434809-centimeter water (4°C).
10-millimeter mercury (0°C) = 13.3322 hectopascals.
10-centimeter water (4°C) = 9.80638 hectopascals.

## Figures and Tables

**Table 1:** Group of children with destruction of the microbubbles in the urinary bladder during ce-VUS examination.

Number	Sex	Age (years months)	ce-VUS Reflux Right Kidney	ce-VUS Reflux Left Kidney	Urinary bladder max voiding vol. (mL)	*T*1 (min)	*T*2 (min)	*T*3 (min)	Anxiety (A); crying (C); multiple voiding attempts (V)
1	M	9 y 3 m	F-G0; V-N/A	F-G0; V-N/A	260	2	4	14	V
2	F	7 y 2 m	F-G0; V-N/A	F-G0; V-N/A	190	10	12	16	V
3	M	1 y 9 m	F-GI; V-N/A	F-G0; V-N/A	100	6	6	20	A; C
4	M	2 y 8 m	F-G0; V-N/A	F-G0; V-N/A	130	4	5	11	A; C
5	M	8 y 1 m	F-GIII; V-N/A	F-GII; V-N/A	300	4	6	12	V
6	M	11 m	F-GIV; V-N/A	F-GIV; V-N/A	50	5	5	8	A; C
7	M	2 y	F-GIII; V-N/A	F-GIV; V-N/A	70	4	6	22	A; C
8	F	6 m	F-G0; V-N/A	F-G0; V-N/A	50	2	4	9	A; C
9	M	2 y 2 m	F-GII; N/A	F-GIII; N/A	100	3	3	16	A; C
10	F	4 m	F-GIII; N/A	F-GII; N/A	40	2	4	10	A; C
11	F	1 y 5 m	F-G0; V-N/A	F-G0; V-N/A	90	3	4	8	A; C

Sex = female (F) and male (M).

Reflux Right Kidney = filling phase (F); voiding phase (V); Grades I–V (GI–V); no reflux (G0); not possible to estimate due to destruction of microbubbles (N/A).

Reflux Left Kidney = filling phase (F); voiding phase (V), Grades I–V (GI–V), no reflux (G0); not possible to estimate due to destruction of microbubbles (N/A).

*T*1: time of the urinary bladder filling.

*T*2: first symptoms of destruction.

*T*3: subtotal/total destruction.

**Table 2:** Technical details concerning ce-VUS (results are given as median (min–max) values).

	Cases	Controls
*N*	11	28
Age	2.1 (4 m–9 y)	2.0 (1 m–11 y)
Sex (% female)	36.4	39.3
Bladder filling time (min)	4.0 (2–10)	4.0 (0.50–5)
Total examination time (min)	12.0 (8–22)	5.50 (4.30–10)
Destruction of microbubbles (min)	5.0 (4–12)	—
